# Correction to “Robot‐Assisted Partial Nephrectomy via a Retroperitoneal Approach With Selective Arterial Clamping Guided by Blood Flow Analysis in a Horseshoe Kidney: A Case Report”

**DOI:** 10.1002/iju5.70184

**Published:** 2026-04-21

T. Fujita, K. Kobayashi, G. Hayashi, S. Kamijo, and F. Ito, “Robot‐Assisted Partial Nephrectomy via a Retroperitoneal Approach With Selective Arterial Clamping Guided by Blood Flow Analysis in a Horseshoe Kidney: A Case Report,” *IJU Case Reports* 9, no. 3 (2026): e70178, https://doi.org/10.1002/iju5.70178.

In the published article, there was an error in the description of the patient's position.

In the “Case Presentation” section, the text “Surgery was performed under general anesthesia with the patient in the right lateral decubitus position.” was incorrect.

This should have read: “Surgery was performed under general anesthesia with the patient in the left lateral decubitus position.”

This correction does not affect the results or conclusions of the article.

We apologize for this error.

